# Post-Irradiation Morphea in Breast Cancer: An Uncommon Differential Diagnosis to Keep in Mind

**DOI:** 10.4021/wjon264w

**Published:** 2011-01-01

**Authors:** Francisco Javier Afonso-Afonso, Maria Pilar Arevalo, Fernando Campo Cerecedo, Laura De Paz Arias, Cristina Durana Tonder

**Affiliations:** a Medical Oncology Section, Complejo Hosp. A. Marcide, Ferrol, A Coruna, Spain; b Dermatology Service, Complejo Hosp. A Coruna, A Coruna, Spain; c Pathology Service, Complejo Hosp. A. Marcide, Ferrol, A Coruna, Spain

**Keywords:** Morphea, Breast cancer, Radiotherapy

## Abstract

Breast cancer is the most common cancer in women of developed countries. Early stage diagnosis is followed, in many cases, of conservative surgery and local radiotherapy. This treatment reduces loco-regional recurrences but may be accompanied by local complications. Morphea of the breast is an uncommon skin condition and has been described after radiotherapy. The inflammatory stage of morphea can commonly be mistaken for a local recurrence or inflammation. We report the case of a 51-year-old woman with breast cancer who underwent a conservative surgery with postoperative chemo and radiotherapy. She developed a sclerodermiform plaque with a biopsy that showed histopathological changes consistent with morphea.

## Introduction

Morphea is a localized scleroderma variety that can be divided into localized (circumscribed) or generalized disease, and is characterized by patches of sclerotic skin that develop on the trunk and limbs at sites of previously normal texture, preferably in the trunk of women of medium age. During the last decade, some cases of radiotherapy related morphea has been reported in breast cancer patients, with great clinical similarity between this entity and a local recurrence. Thus, it is a very important issue to keep in mind this idiopathic disease in the differential diagnosis of a breast cancer patient with a probably local post-radiotherapy recurrence.

## Case Report

**Figure 1:**
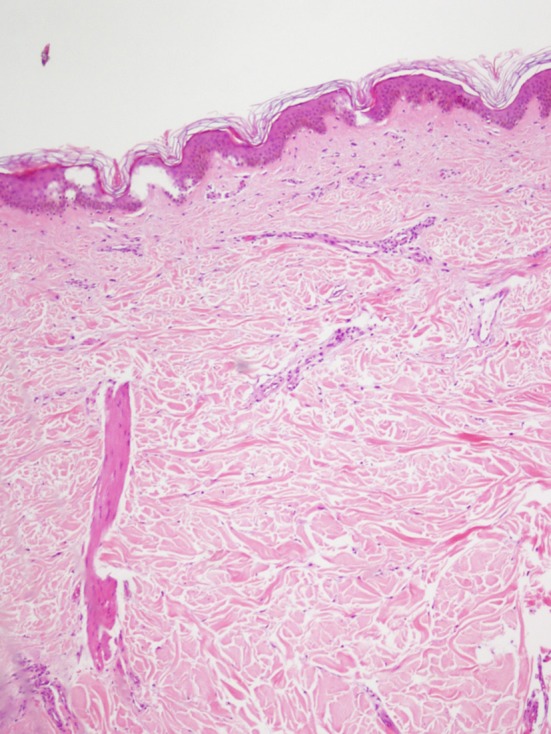
Dermis with numerous thickened collagen fibers parallel to the basal layer and atrophy of skin appendages (HE x 100).

A 51-year-old post-menopause Caucasian woman was diagnosed with right breast carcinoma. Histopathological diagnosis was made from a needle biopsy: invasive ductal adenocarcinoma, with positivity for estrogen and progesterone receptors and negative C-erbB2. A wide local excision of the right breast and axillary node sampling was realized and post operatory histology identified a T1N1 grade 2 invasive ductal carcinoma without margins invasion. She was treated with adjuvant chemotherapy using four cycles of doxorubicin and ciclofosfamide followed by four cycles of docetaxel. Letrozole 2.5 mg every day was taken when chemotherapy was concluded and conventional external radiotherapy to the right breast, axillary and internal mammary nodes were indicated, with a total dose of 50 Gy. Eleven months after the radiotherapy end, patient developed a pruritic plaque of gradual growth located in the radiotherapy field on the right breast. Mammography and ultrasound scan demonstrated a thickness of the cellular subcutaneous tissue and a density increase of the underlying fat. Office inspection showed an irregular indurate plaque, with irregular borders and “a peau d´orange” aspect. A biopsy revealed non epidermis changes and dermis thickness with numerous thick of collagen fibers that in depth were penetrating into the fatty lobules of the cellular subcutaneous tissue, a slight inflammatory infiltrate, and an atrophy of pilocebaceous units, consistent with a diagnosis of localized scleroderrmia (morphea) (Fig. 1). Patient was treated with topical local steroids, with progressive improvement and without new loco-regional dermatological events.

## Discussion

In developed countries, breast cancer is the most frequent neoplasm in women. Conservative surgery, followed by local radiotherapy is a common modality used in the treatment of this disease. This treatment reduces loco-regional recurrences but may be accompanied by local acute (erythema and desquamation) or chronic fibrosis (10%), skin telangiectasia (10%), atrophy (8%), and pain (2%) [1]. Morphea is a localized scleroderma variety that can be divided into localized (circumscribed) or generalized disease, and is characterized by patches of sclerotic skin that develop on the trunk and limbs at sites of previously normal texture, preferably in the trunk of women of medium age [2]. Morphea post-radiotherapy is a rare complication, with an estimated incidence of 1 in 500 patients [3], in contrast to that of morphea (of any etiology), which is 2.7 per 100 000 in the general population per year [4], and its onset ranges from 1 to 12 months, but can be as late as 32 years post-radiotherapy [5, 6]. Today, we know that there is no relationship with the total radiotherapy dose [7]. All the involved pathogenic mechanisms are not known, but it is accepted that radiotherapy-induced morphea has an initial inflammatory phase and a subsequent 'burnt-out' phase, where the latter displays induration, fibroid retraction, and pigmentation of the breast. The pathophysiology is thought to be radiation-induced neoantigen formation that subsequently stimulates secretion of transforming growth factor beta (TGF-β). TGF-β strongly induces fibroblast activation, collagen synthesis, and, hence, excessive fibrosis [5, 6]. Usually, the clinical course of radiotherapy-induced morphea is benign and it will usually improve with any of the many treatments that have been used with varying results, including oral and topical antibiotics, intralesional or systemic steroids, oral antimalarial drugs, or phototherapy [5]. Some publications suggest that radiotherapy alone most likely is not sufficient to provoke morphea and surgery is an important but as yet underestimated risk factor in disease etiogenesis [8]. Hence, probably a synergistic pathogenic effect of both treatment modalities in a subset of patients with particular immunologic characteristics triggers the manifestation of this entity. During the last decade, some cases of radiotherapy related morphea has been reported in breast cancer patients, with great clinical similarity between this entity and a local recurrence. Thus, it is a very important issue to keep in mind this idiopathic disease in the differential diagnosis of a breast cancer patient with a probably local post-radiotherapy recurrence.
